# Transcriptional profile of *Salmonella enterica* subsp. *enterica* serovar Weltevreden during alfalfa sprout colonization

**DOI:** 10.1111/1751-7915.12104

**Published:** 2013-12-06

**Authors:** Kerstin Brankatschk, Tim Kamber, Joël F Pothier, Brion Duffy, Theo H M Smits

**Affiliations:** Plant Protection Division, Agroscope Changins-Wädenswil ACWSchloss 1, Wädenswil, CH-8820, Switzerland

## Abstract

Sprouted seeds represent a great risk for infection by human enteric pathogens because of favourable growth conditions for pathogens during their germination. The aim of this study was to identify mechanisms of interactions of *Salmonella enterica* subsp. *enterica* Weltevreden with alfalfa sprouts. RNA-seq analysis of *S*. Weltevreden grown with sprouts in comparison with M9-glucose medium showed that among a total of 4158 annotated coding sequences, 177 genes (4.3%) and 345 genes (8.3%) were transcribed at higher levels with sprouts and in minimal medium respectively. Genes that were higher transcribed with sprouts are coding for proteins involved in mechanisms known to be important for attachment, motility and biofilm formation. Besides gene expression required for phenotypic adaption, genes involved in sulphate acquisition were higher transcribed, suggesting that the surface on alfalfa sprouts may be poor in sulphate. Genes encoding structural and effector proteins of *Salmonella* pathogenicity island 2, involved in survival within macrophages during infection of animal tissue, were higher transcribed with sprouts possibly as a response to environmental conditions. This study provides insight on additional mechanisms that may be important for pathogen interactions with sprouts.

## Introduction

Outbreaks of zoonotic pathogens like *Salmonella* serovars or *Escherichia coli* O157:H7 are commonly known to be linked to meat products from bovine, pork or poultry (Chiu *et al*., 2005). Increasingly, outbreaks associated with contaminated sprouts and fresh vegetable produce (e.g. lettuce, spinach, tomato) are becoming a public health concern (Taormina *et al*., 1999; Sivapalasingam *et al*., 2004; Berger *et al*., 2010). A possible explanation is increased consumption caused by enhanced recognition by the broader public of sprouts as nutritious food. However, during mass production thereof, favourable conditions are generated during germination for bacteria such as *Salmonella* spp., especially when hygienic standards are not followed (Studer *et al*., 2013). In Europe, outbreaks linked to contaminated sprouts were caused by *S. enterica* subsp. *enterica* serovars Stanley, Bovismorbificans and Bareilly (Cleary *et al*., 2010). Another *Salmonella* serovar, *S. enterica* subsp. *enterica* serovar Weltevreden, that is commonly known to be a problem associated with meat products in Southeast Asia (Sood and Basu, 1979; Bangtrakulnonth *et al*., 2004; Learn-Han *et al*., 2008) recently emerged in Western countries, linked not only to meat but also to vegetable products. This serovar was recognized for the first time on plant products as the cause of an outbreak of gastroenteritis in Scandinavia (Norway, Denmark and Finland) resulting from consumption of contaminated alfalfa sprouts (Emberland *et al*., 2007). This outbreak was caused by seeds contaminated with *S.* Weltevreden that regrew during germination (Taormina *et al*., 1999; Emberland *et al*., 2007).

During epidemiological investigations, seeds were found to be the source of several outbreaks. Isolation of *Salmonella* spp. from sprouts and their seeds suggests that enteric pathogens can colonize, multiply and persist for prolonged periods of time during production of sprouts. For contamination, only minimal levels of *Salmonella* spp. are necessary, as the pathogens can multiply fast during the manufacturing processes with sprouts. Despite optimal growth conditions for enteric pathogens with sprouts, only *Salmonella* spp. and *E. coli* O157:H7 have been isolated so far. Therefore, colonization mechanisms that are active during interactions with sprouts are of great interest to explain enhanced detection of these pathogens. In a comparative experiment, it was shown that *Salmonella* spp. can attach significantly better to sprouts than *E*. *coli* O157:H7 (Barak *et al*., 2002). In another study of the same group, it was found that certain virulence genes are necessary for attachment of plant tissue (Barak *et al*., 2005). Mutants of *agfB* (also named *csgD*), a surface-exposed aggregative fimbria nucleator (Nuccio and Baumler, 2007) that regulates curli and cellulose production, and of *rpoS*, regulating the same and other adhesins such as pili, showed reduced adherence to alfalfa sprouts. Upregulation of flagellar regulons and fimbrial genes were also found for *E. coli* O157:H7 during growth on lettuce lysate (Kyle *et al*., 2010). Besides genes responsible for motility and attachment, genes involved in carbohydrate metabolism and stress responses, genes encoding pathogenicity islands (LEE operons) and putative effector proteins were also upregulated in lettuce lysate (Kyle *et al*., 2010).

In the genome of *S*. Weltevreden 2007-60-3289-1, a strain isolated after an outbreak in Scandinavia in association with alfalfa sprouts, we found three serovar-specific genomic islands (GIs), encoding carbohydrate metabolism genes (Brankatschk *et al*., 2012). Analysis by reverse transcription-polymerase chain reaction (RT-PCR) showed that only genes of GI_VI encoding proteins putatively involved in mannitol degradation were transcribed with sprouts. Additionally, we found that *S*. Weltevreden 2007-60-3289-1 was able to grow on additional stereoisomers of *myo*-inositol, a carbohydrate ubiquitous distributed in the environment such as on plants. The additional carbohydrate clusters and possibility to utilize more than one stereoisomer of *myo*-inositol might enhance survival of this serovars on plants.

By analysing the complete transcriptome of *Salmonella* spp. on vegetables, our study aimed to identify genes that are differentially regulated during growth with sprouts in comparison to growth in a minimal medium without bacterial competition. To cover the complete transcriptome, enriched mRNA from both growth conditions was analysed by RNA-seq, the analysis of steady state RNA using next generation sequencing techniques (Wilhelm *et al*., 2008; Passalacqua *et al*., 2009; Wang *et al*., 2009; Raabe *et al*., 2011). To verify results of RNA-seq analysis, a number of genes that had a higher transcription level in presence of sprouts were chosen to be analysed using quantitative RT-PCR (qRT-PCR). Analysis was done with sprout samples as well as with leafy salad, spinach and lamb's lettuce.

## Results

**Fig 1:**
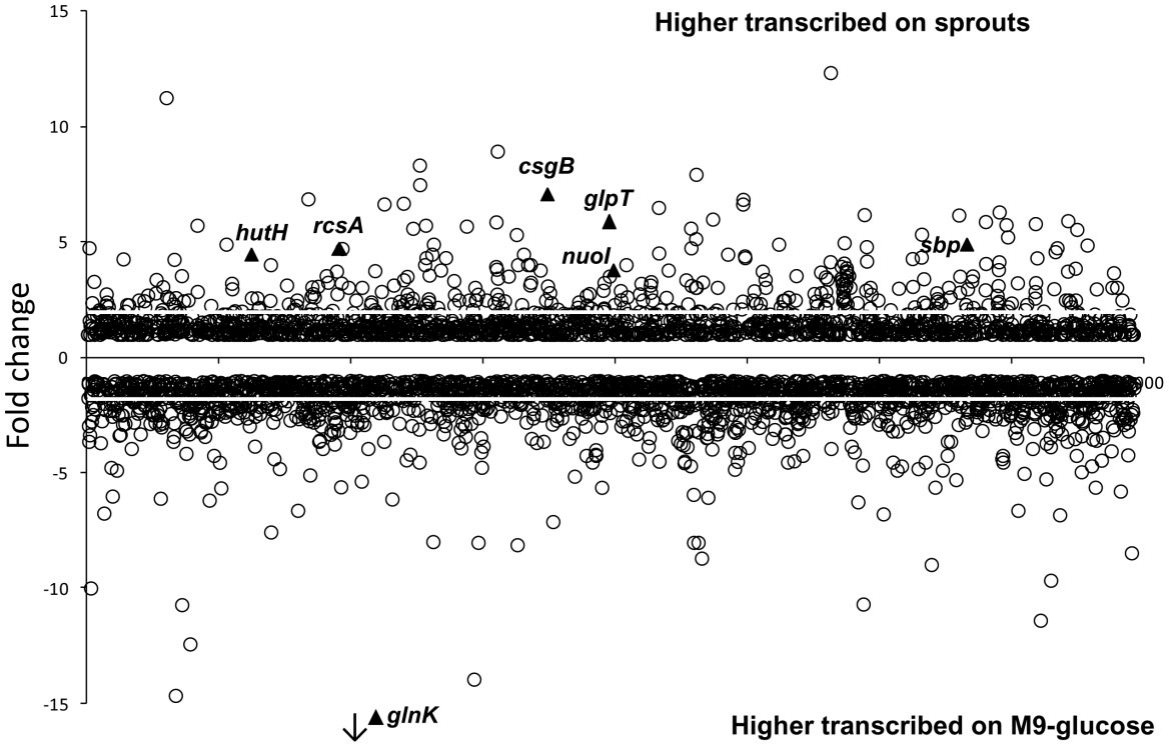
Fold change of genes higher transcribed during growth with sprouts in comparison with growth in M9-glucose medium. A negative fold change shows higher expression of genes in M9-glucose medium whereas a positive fold change shows higher expression in presence of sprouts. Altogether, 4158 genes were compared using Cufflinks whereas expression of 522 genes was significantly different (*P* < 0.05) with a fold change higher than 2.0 or lower than −2.0 (white line). Genes indicated as triangle and labelled with a gene name were used for qRT-PCR. As the fold change for *glnK* is out of scale while it is only transcribed in M9-glucose medium, it is indicated with an arrow.

*Salmonella* Weltevreden 2007-60-3289-1 was grown with sprouts and in M9-glucose medium and harvested in the mid-exponential growth phase. RNA-seq analysis of these samples resulted in expression signals for 4158 genes. About 522 genes (12.55%) were significantly differential transcribed between both growth conditions, of which 177 (4.267%) were more transcribed in presence of sprouts and 345 (8.30%) were more transcribed in M9-glucose medium (Fig. 1; Table 1). Altogether, 14 genes were not transcribed in presence of sprouts but were transcribed in M9-glucose medium, whereas no genes were only transcribed in presence of sprouts.

**Table 1:** Genes higher transcribed in presence of sprouts in comparison to M9-glucose medium determined by RNA-seq analysis

Category	Gene	Locus tag	Fold change	Function
Amino acid metabolism	*argI*	SENTW_4577	11.84	Ornithine carbamoyltransferase 1
	*speB*	SENTW_3201	5.79	Agmatinase
	*speD*	SENTW_0130	8.12	S-adenosylmethionine decarboxylase
	*putA*	SENTW_1034	5.07	Transcriptional repressor, proline oxidase
	*putP*	SENTW_1045	5.01	Sodium/proline symporter
	*ilvB*	SENTW_3900	5.34	Acetolactate synthase
	*ilvN*	SENTW_3899	8.33	Acetolactate synthase small subunit
	*avtA*	SENTW_3763	4.67	Valine pyruvate aminotransferase
	*phnA*	SENTW_4376	9.47	Alkylphosphonate utilization operon protein
	*hutH*	SENTW_0770	8.68	Histidine ammonia-lyase
	*hutU*	SENTW_0769	3.93	Urocanate hydratase
	*carA*	SENTW_1700	9.47	Carbamoyl-phosphate synthase small chain
	*sbp*	SENTW_4153	9.94	Sulphate-binding protein
	*cysM*	SENTW_2618	4.46	Cysteine synthase B
	*cysI, cysJ*	SENTW_3043-42	8.26	Sulphite reductase
	*cysH*	SENTW_3041	8.47	Sulphate reductase
	*cysD*	SENTW_3022	16.05	Sulphate adenylsltransferase subunit
	*cysC*	SENTW_3020	15.37	Adenosine 5′-phosphosulphate kinase
	*cysN*	SENTW_3021	15.37	Sulphate adenylsltransferase subunit
	*cysA, cysW, cysT, cysP*	SENTW_2620-23	5.56	Sulphate transporter
	*metB*	SENTW_4189	5.63	Cystathionine gamma-synthase
	*ST2*	SENTW_4354	5.43	Sulphate transporter
	*pphA*	SENTW_1338	311.10	Serine/threonine-protein phosphatase
Pathogenicity island	*sifA*	SENTW_2029	8.68	Secreted protein
(SPI-2)	*ssaG, ssaH, ssaI*			
	*ssaJ, ssaK, ssaL*	SENTW_1805-011	4.29	Secretion apparatus
	*ssaT, ssaU*	SENTW_1794-95	7.22	Secretion apparatus
	*ssaM, ssaV*	SENTW_1800-01	6.91	Secretion apparatus
	*sseE*	SENTW_1812	5.93	Effector protein
	*sscB*	SENTW_1811	23.57	Chaperone
	*ssaR*	SENTW_1796	12.86	Export apparatus
	*sopD2*	SENTW_3040	5.99	Effector protein
	*pipB*	SENTW_1007	4.01	Effector protein
	*sseL*	SENTW_2415	5.46	Deubiquitinase
Motility	*csgB*	SENTW_2110	16.75	Minor curli subunit
	*csgG*	SENTW_2114	5.49	Curli production assembly/transport component
	*csgD*	SENTW_2111	5.21	Transcriptional regulator
	*bcfE*	SENTW_4730	4.81	Fimbrin-like protein FimI
	*spy*	SENTW_1904	6.15	Spheroplast protein
Cofactors and energy production	*thiC, thiE, thiF, thiS*	SENTW_4269-72	12.48	Thiamin phosphate pyrophosphorylase, Thiamine biosynthesis proteins
	*nuoI*	SENTW_2443	6.82	NADH dehydrogenase I (chain I)
	*nuoE, nueF*	SENTW_2449-50	3.30	NADH dehydrogenase I (chains E and F)
	*metF*	SENTW_4195	6.17	Methylenetrahydrofolate reductase
	*atpG*	SENTW_3971	4.42	Membrane-bound ATP synthase
Regulators	*fis*	SENTW_3516	37.55	DNA binding protein
	*yiaG*	SENTW_3750	13.86	Transcriptional regulator
	*metR*	SENTW_4054	13.81	Transcriptional regulator
	*rcsA*	SENTW_1101	9.30	Regulator of capsular polysaccharide synthesis
	*ydcI*	SENTW_1576	6.26	Probable RuBisCO transcriptional regulator
	*ydhM*	SENTW_1780	5.67	HTH-type transcriptional repressor
	*ydcN*	SENTW_1600	4.29	Uncharacterized HTH-type transcriptional regulator
	*mntR*	SENTW_0817	3.92	Manganese transport regulator
Stress response	*pspA*	SENTW_1509	15.49	Phage shock protein
	*pspB, pspC*	SENTW_1510-11	5.75	Phage shock protein
	*ibpA*	SENTW_3916	5.86	Heat shock protein
	*osmY*	SENTW_4668	9.81	Osmotically-inducible protein
Transporters	*yehW*	SENTW_1718	8.27	Bicarbonate transport system permease
	*fliY*	SENTW_1129	5.49	Cysteine-binding periplasmic protein
	*yliA*	SENTW_0829	2.92	Glutathione transporter
	*corA*	SENTW_4042	4.96	Magnesium transporter protein
	*dctA*	SENTW_3716	4.87	C4-dicarboxylate transport protein
	*ybiR*	SENTW_0818	2.58	Inner membrane protein
	*ydjN3*	SENTW_1892	12.28	L-cystine uptake protein tcyP
Protein export; Bacterial secretion system	*yajC*	SENTW_0393	4.06	Preprotein translocase subunit YajC
	*uraA*	SENTW_2680	14.89	Uracil permease
Lipid metabolism	*glpK*	SENTW_4175	12.90	Glycerol kinase
	*glpT*	SENTW_2411	12.82	Glycerol-3-phosphate transporter
	*glpF*	SENTW_4176	7.58	Glycerol uptake facilitator protein
	*glpQ*	SENTW_2410	6.15	Glycerophosphodiester phosphodiesterase
	*cdh1*	SENTW_4154	4.81	CDP-diacylglycerol pyrophosphatase
	*yjfO*	SENTW_4482	13.03	Lipoprotein
	*ybaY*	SENTW_0451	6.20	Uncharacterized lipoprotein
Fatty acid metabolism	*fadB*	SENTW_4073	6.14	Enoyl-CoA hydratase
	*fadA*	SENTW_4072	4.47	Small (beta) subunit of the fatty acid-oxidizing multienzyme complex
Post-transcriptional modification	*queA*	SENTW_0390	11.26	Synthesis of queuine in tRNA
	*trmD*	SENTW_2838	9.46	tRNA methyltransferase
	*yhdG*	SENTW_3515	7.78	tRNA-dihydrourindine synthase B
	*rpoA*	SENTW_3542	7.24	DNA-dependent RNA polymerase
Carbohydrate metabolism	*aceB*	SENTW_4287	10.86	Malate synthase A
	*acs*	SENTW_4361	8.99	Acetyl-coenzyme A synthetase
	*sdhC, sdhD, sdhA*	SENTW_0709-11	5.39	Succinate dehydrogenase
	*sdhB*	SENTW_0712	4.82	Succinate dehydrogenase iron-sulphur protein
	*sucC,sucD*	SENTW_0716-17	2.63	Succinyl-CoA synthetase
	*prsA*	SENTW_1416	5.20	Ribose-Phosphate pyrophosphokinase
	*cpsB, rfbK*	SENTW_2210-11	3.06	Mannose-1-phosphate guanylyltransferase; phosphomannomutase
Nucleotide metabolism	*upp*	SENTW_2681	8.83	Uracil phosporibosyltransferase
	*nrdA*	SENTW_2405	4.66	Ribonucleoside-diphosphate reductase alpha
Genetic information processing –				
Replication and repair	*priB*	SENTW_4495	4.54	Primosomal replication protein N
	*ruvC*	SENTW_1185	4.31	Cross-over junction endodeoxyribonuclease
	*rplB*	SENTW_3564	6.05	50S ribosomal protein L2
	*rplD, rplW*	SENTW_3565-66	6.59	50S ribosomal protein L4; 50S ribosomal protein L23
	*rplF*	SENTW_3552	7.62	50S ribosomal protein L6
	*rplI*	SENTW_4497	5.45	50S ribosomal protein L9
	*rplJ*	SENTW_4260	7.77	50S ribosomal protein L10
	*rplK*	SENTW_4258	6.87	50S ribosomal protein L11
	*rplL*	SENTW_4261	14.24	50S ribosomal protein L7/L12
	*rplO*	SENTW_3548	4.54	50S ribosomal protein L15
	*rplP, rpmC, rpsQ*	SENTW_3558-60	5.73	50S ribosomal protein L16; 50S ribosomal protein L29; 30S ribosomal protein S17
	*rplQ*	SENTW_3541	5.94	50S ribosomal protein L17
	*rplS*	SENTW_2837	12.01	50S ribosomal protein L19
	*rplU*	SENTW_3432	4.42	50S ribosomal protein L21
	*rplV*	SENTW_3562	6.73	50S ribosomal protein L22
	*rpmA*	SENTW_3431	6.60	50S ribosomal protein L27
	*rpmB*	SENTW_3829	5.06	50S ribosomal protein L28
	*rpmD*	SENTW_3549	3.82	50S ribosomal protein L30
	*rpmF*	SENTW_2062	6.82	50S ribosomal protein L32
	*rpmG*	SENTW_3828	8.15	50S ribosomal protein L33
	*rpmJ*	SENTW_3546	5.27	50S ribosomal subunit protein L36
	*rpsH*	SENTW_3553	10.11	30S ribosomal protein S8
	rpsI	SENTW_3473	6.06	30S ribosomal protein S9
	rpsK	SENTW_3544	5.67	30S ribosomal protein S11
	rpsR	SENTW_4496	12.63	30S ribosomal protein S18
	rpsS	SENTW_3563	7.07	30S ribosomal protein S19
	rpsT	SENTW_4750	6.53	30S ribosomal protein S20
	rpsU	SENTW_3346	9.92	30S ribosomal protein S21
	yfjA	SENTW_2839	5.16	Ribosome maturation factor RimM
Unclassified	*yaiB*	SENTW_0366	5.03	Anti-adapter protein IraP
	*nusG*	SENTW_4257	5.15	Elongation factor
	*rnt*	SENTW_1783	4.24	Ribonuclease T
	*cesT*	SENTW_2267	4.16	Putative cytoplasmic protein
	*sixA*	SENTW_2511	4.02	Phosphohistidine phosphatase
	*rnpA, yidD*	SENTW_3942-43	3.26	RNase P, protein component
	*era, rnc*	SENTW_2769-70	2.63	GTP-binding protein era homolog
	*ntpA*	SENTW_1183	6.70	dATP pyrophosphohydrolase
	*ydcF*	SENTW_1578	8.27	Putative esterase
Hypothetical proteins		SENTW_0391	32.88	Hypothetical protein
	*ycdZ*	SENTW_2115	5.11	Hypothetical protein
		SENTW_1535	21.31	Hypothetical protein
		SENTW_1536	18.25	Hypothetical protein
		SENTW_1962	11.16	Hypothetical protein
		SENTW_1381	6.75	Hypothetical protein
	*yeeI*	SENTW_2127	4.43	Hypothetical protein
	*yibP*	SENTW_3805	4.27	Hypothetical protein
	*yigM*	SENTW_4053	4.01	Hypothetical protein
		SENTW_0384	3.95	Hypothetical protein
	*sopD*	SENTW_0915	13.24	Homologous to secreted protein SopD (T3SS)
	*yahO*	SENTW_0348	4.40	Protein of unknown function
	*yebV*	SENTW_1340	15.38	Uncharacterized protein
	*yiaK*	SENTW_3767	9.61	Putative protein
	*yciF*	SENTW_1469	11.99	Unknown function
	*ygaT (csiD)*	SENTW_2877	19.85	Hypothetical protein
	*tctA*	SENTW_2876	10.62	Unknown function
	*yqeF*	SENTW_3132	4.61	Putative acyltransferase
	*TPX*	SENTW_1534	6.12	Putative thiol peroxidase
	*ybdL*	SENTW_0580	4.55	Putative aminotransferase
	*yjgF*	SENTW_4564	7.03	Protein TdcF
	*yiaL*	SENTW_3768	7.86	Protein YiaL
	*ytfK*	SENTW_4510	8.24	Uncharacterized protein YtfK
	*yhcN3*	SENTW_3490	5.82	Protein YdgH
	*ygaU*	SENTW_2883	5.51	Uncharacterized protein YgaU
	*yggE*	SENTW_3181	6.70	Uncharacterized protein YggE
	*yfeK*	SENTW_2617	5.38	Uncharacterized protein YfeK
	*yjfN*	SENTW_4481	6.70	UPF0379 protein YjfN

### Genes more transcribed in presence of sprouts

**Fig 2:**
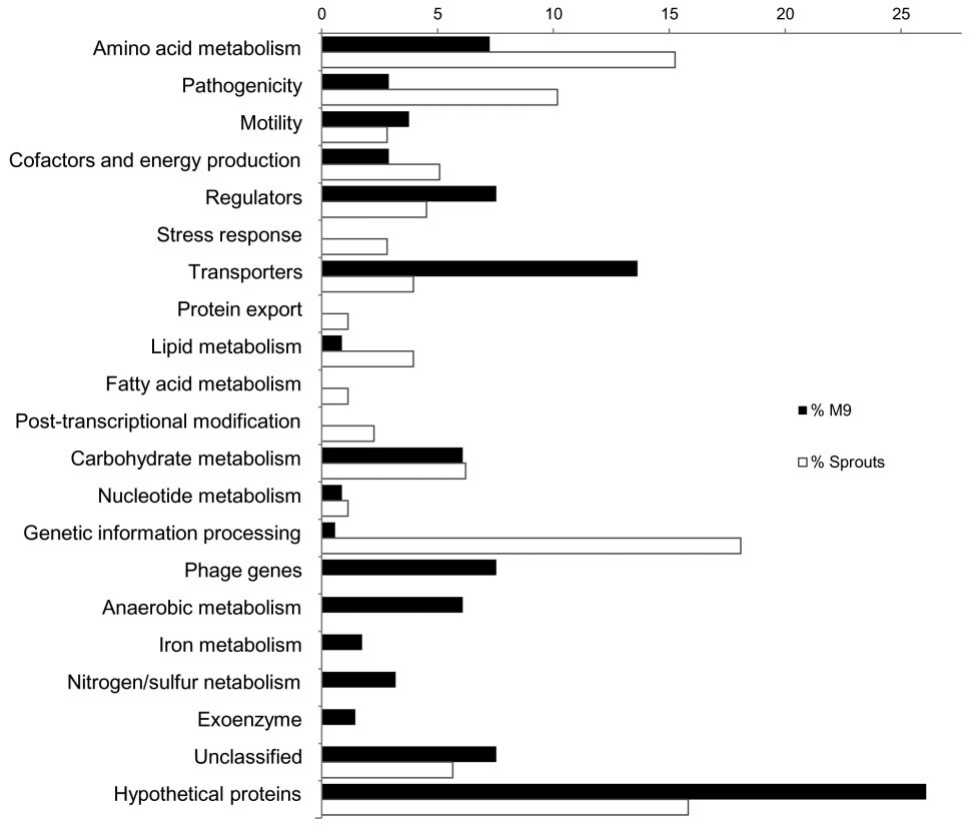
Relative percentage of genes significantly more transcribed during growth in presence of sprouts (white bars) compared with M9-glucose medium (black bars). Functions of genes of interest were classified according to the Kyoto Encyclopedia of Genes and Genomes pathway database.

According to Kyoto Encyclopedia of Genes and Genomes (KEGG) categorization, genes significantly more transcribed in presence of sprouts include genes involved in amino acid metabolism, carbohydrate metabolism, genetic information processing and *Salmonella* infection [*Salmonella* pathogenicity island (SPI)-2; Fig. 2 ]. Various genes remained unclassified as they encode hypothetical proteins or proteins with an unknown function. A major difference is that around 30 ribosomal proteins are significantly higher transcribed in presence of sprouts than in M9-glucose medium (Table 1). This difference might be caused by a different growth rate in the two conditions. As the growth with sprouts could not be quantified because of biofilm formation, quantitative differences are not given.

### Sulphate/cysteine biosynthesis and acquisition with sprouts

**Fig 3:**
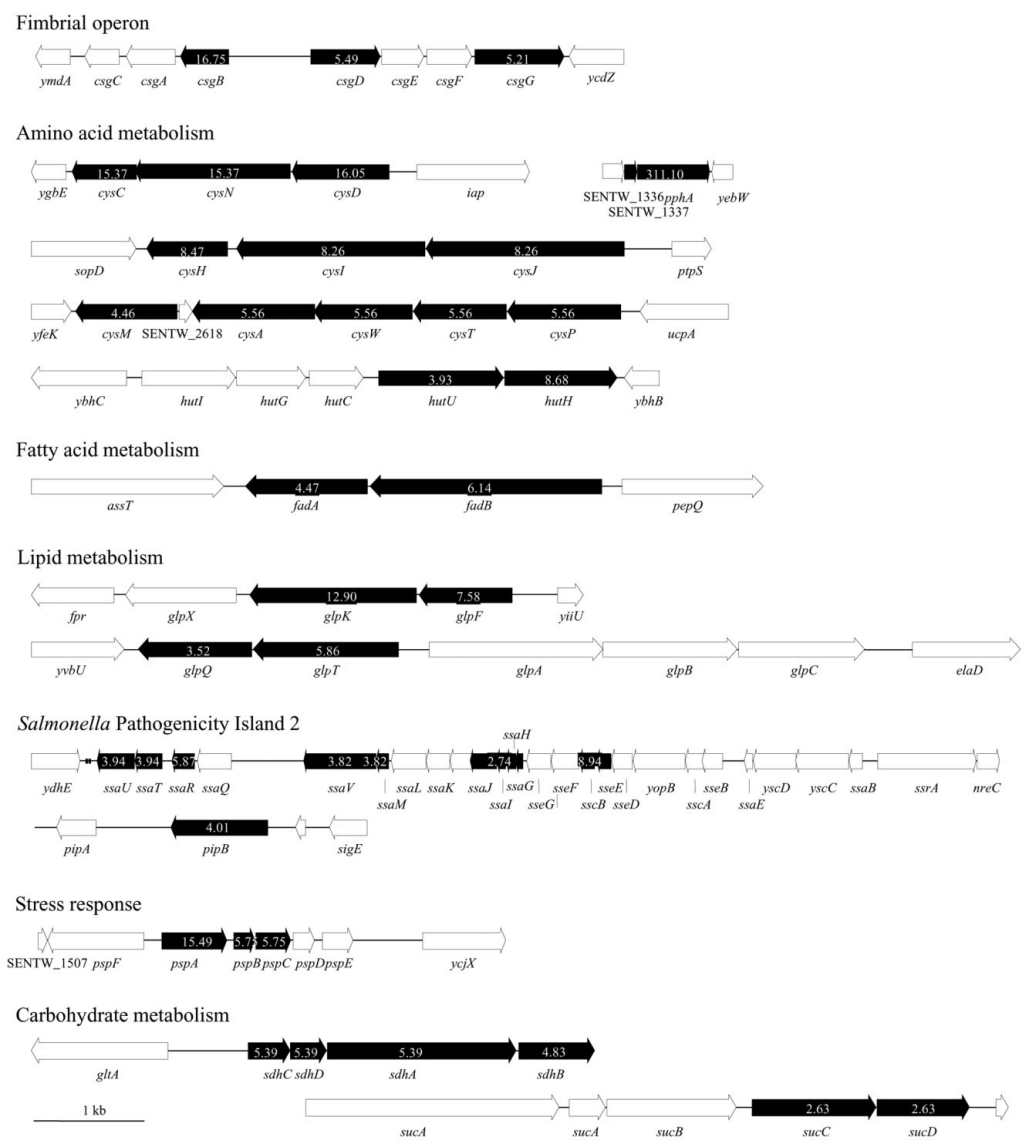
Representative gene clusters of genes with higher transcription in presence of sprouts compared with M9-glucose medium. Genes with increased expression in presence of sprouts are presented as black arrows. Numbers inside arrows indicate the fold change between growth in M9-glucose medium and with sprouts as determined by RNA-seq. Genes having no significant difference in transcription level are indicated in white.

Altogether, 21 genes were more transcribed in presence of sprouts encoding proteins involved in amino acid metabolism which represented the cluster with most genes significantly more transcribed in presence of sprouts in one category. Of these 21 genes, 12 genes encode part of cysteine biosynthesis and acquisition (Fig. 3, Table 1). Two uptake and reduction systems for sulphate were upregulated in presence of sprouts. Almost all genes encoding genes necessary for reduction of sulphate (*cysD* and *cysN*; SENTW_3022 and 3021) over sulphite (*cysHC;* SENTW_3041, 3020) to sulphide (*cysIJ;* SENTW_3043-42) were more transcribed in presence of sprouts after extracellular sulphate entered the cell via a sulphate-binding protein encoded by *spb* (SENTW_4153) and a sulphate permease (*cysAW;* SENTW_2620-21). The genes encoding proteins involved in sulphate uptake (*cysAW*) can also transport external thiosulphate into the cell and attach O-acetylserine (thiol)-lyase to *S*-sulphocysteine (*cysM*; SENTW_2618), which is later transformed to cysteine (Sekowska *et al*., 2000). The gene encoding MetB, the cystathionine gamma-synthase (Sekowska *et al*., 2000), which plays a role in methionine synthesis, was transcribed higher in presence of sprouts than in M9-glucose medium. Other single genes involved amino acid utilization pathways like degradation of histidine [*hutH*, *hutU* (SENTW_0769–0770)], arginine and orthinine (*argI, speB, speD*), arginine and proline (*putA, putP*), valine, leucine and isoleucine (*ilvB, ilvN, phnA, avtA*) were more transcribed in presence of sprouts. Most of these proteins are involved in multiple pathways or catalyse more than one step in the amino acid metabolic pathway.

### Fimbrial genes

In response to sprouts, genes encoding curli involved in adhesion to surfaces, cell aggregation and biofilm formation were more transcribed. The gene encoding CsgA, the major curli subunit, was transcribed, but not significantly higher in presence of sprouts, whereas *csgB* (SENTW_2110), encoding the anchor for curli fibre which is composed of polymerized monomers (Loferer *et al*., 1997), was more transcribed in presence of sprouts (Table 1, Fig. 3). Similarly, *csgG* and *csgD* (SENTW_2114, 2111) which are part of the *csgDEFG* operon encoding accessory proteins that facilitate the secretion and assembly of CsgA into a fibre were higher transcribed. Another gene encoding a fimbrin-like protein, *bcfE* (SENTW_4730), which was found in *E. coli* to play a role in pilus biosynthesis (Valenski *et al*., 2003), was more transcribed in presence of sprouts.

### Type III secretion systems

One of the major virulence factors of *Salmonella* is the type III secretion system (T3SS) located on SPI-2, involved in survival in macrophages during animal infection (Cirillo *et al*., 1998). Genes encoding structural and effector proteins thereof were more transcribed in presence of sprouts, including those encoding part of the secretion apparatus SsaGHIJ (SENTW_1805-08), SsaM, SsaR and SsaTUV (SENTW_1794,1795, 1796, 1800 and 1801) as well as a chaperone (*sscB*; SENTW_1811). Additionally, five genes encoding effector proteins [SifA, SseE, SopD, PipB and SseL (SENTW_2029,1812, 3040, 1007 and 2415)] were more transcribed in presence of sprouts that play a role in pathogen–host interaction by formation of lysosomal glycoprotein-containing structures in epithelial cells (SifA, SENTW_2029), regulation of aggregative fimbriae synthesis and biofilm formation (SopD, SENTW_3040) (Römling *et al*., 1998; Prigent-Combaret *et al*., 2001), localization of *Salmonella-*induced filaments (PipB, SENTW_1007) (Knodler *et al*., 2002), regulation of protein secretion (SseE, SENTW_1812) (Cirillo *et al*., 1998) or fitness enhancement of *S*. Typhimurium during colonization of infected host (SseL; SENTW_2415) (Coombes *et al*., 2006).

### Lipid, fatty acid metabolism and thiamine biosynthesis

*Salmonella* spp. as well as *E*. *coli* are able to use glycerol as a carbon source (Gutnick *et al*., 1969). The glycerol facilitator gene *glpF* (SENTW_4176) was more transcribed in presence of sprouts as well as the glycerol kinase encoded by *glpK* (SENTW_4175), which phosphorylates glycerol to glycerol-3-phosphate (Iuchi *et al*., 1990). Another way to obtain glycerol-3-phosphate for biosynthesis is to hydrolyse glycerophosphodiester in the periplasm, which is encoded by *glpQ* (SENTW_2410) and following transport into the cell by a permease encoded by *glpT* (SENTW_2411). Both genes were more transcribed in presence of sprouts.

For fatty acid utilization as a carbon source, at least five separate operons are involved (Bachmann and Low, 1980). The genes *fadA* (SENTW_4072) and *fadB* (SENTW_4073) of the *fadABC* operon encoding the β-oxidation multi-enzyme complex were transcribed 2.8- and 3.5-fold higher than in M9-glucose medium.

The thiamine biosynthetic pathway is complex and is encoded on three operons and four single gene loci (Begley *et al*., 1999). Four genes that encode part of the thiamine pathway were more transcribed during growth with sprouts. These include *thiF*, which encodes an adenyltransferase (SENTW_4270) and *thiS* encoding a sulphur carrier protein (SENTW_4269). Gene *thiC* (SENTW_4272) encodes a hydroxymethyl pyrimidine synthase involved in pyrimidine biosynthesis and *thiE* (SENTW_4271) is required for linking thiazole and pyrimidine. Presence of intermediate products at different levels might lead to differences in expression ratios for each gene involved in the formation of thiazole.

### Regulators

Altogether, eight regulatory genes were more transcribed in presence of sprouts, with *fis* (SENTW_3516) being the regulatory gene with highest fold expression ratio (38-fold). In *S.* Typhimurium, this DNA-binding protein is involved in coordinating the expression of metabolic, flagella and type III secretion factors especially encoded on SPI-2 (Kelly *et al*., 2004). Full expression of *fis* is required for upregulation of genes encoding secretion apparatus of T3SS and effectors required for invasion of host epithelial cells, for survival in macrophages and synthesis of flagella for motility (Kelly *et al*., 2004). Genes encoding the secretion apparatus of SPI-2 and motility genes were found to be more transcribed in presence of sprouts. The gene *ydcI* (SENTW_1576) encoding a conserved DNA-binding protein is related to stress resistance, and possibly, virulence (Jennings *et al*., 2011) was more transcribed in presence of sprouts. Additional higher transcribed genes in presence of sprouts encode the regulator RcsA (SENTW_1101), which is besides RcsB one of the positive regulators for transcription of capsular polysaccharide synthesis in *E. coli* (Sledjeski and Gottesman, 1995) and YdhM (SENTW_1780), which is a putative TetR-family regulator that mainly regulates biosynthesis of antibiotics, efflux pumps and osmotic stress (Ramos *et al*., 2005).

### Stress

Analysis of the transcriptional profile of *S*. Weltevreden grown with sprouts revealed that genes responding to stress were more transcribed than in M9-glucose medium. The genes *pspA*, *pspB* and *pspC* (SENTW_1509–1511) encoding the phage-shock-protein operon (*psp*) which is responsible for damage repair and maintenance of the proton-motive force of the inner membrane (Darwin, 2005; Kobayashi *et al*., 2007) were more transcribed in presence of sprouts. As transcription of *pspA* is prevented under non-induced conditions and transcription *pspA* increases because of the release of PspA from PspF (Dworkin *et al*., 2000; Darwin, 2005), it can be concluded that *S*. Weltevreden is stressed in presence of sprouts. Indeed, of the *psp* operon, *pspA* was transcribed with highest fold change between the two samples.

Other stress response genes more transcribed in presence of sprouts were *ibpA* (SENTW_3916), encoding a heat shock protein that stabilizes thermally aggregated proteins, in combination with IbpB (Kitagawa *et al*., 2000) and the gene *osmY* (SENTW_4668) encoding an osmotically inducible periplasmic protein (Yim and Villarejo, 1992).

### Genes more transcribed in M9-glucose medium

Genes more transcribed during growth in M9-glucose medium (Table S1) encode proteins encoding phage proteins (SENTW_2536, 2552–2553, 2822–2823), an L-fucose-1-phosphate aldolase (SENTW_3083), a PTS system specific for galactitol (SENTW_3389) that is part of the galactose metabolism and a hypothetical protein (SENTW_1049). Additionally, genes *ccmE-H* (SENTW_2375–2380) encoding a heme chaperone [*ccmE*, (Schulz *et al*., 1998)], a small membrane protein [*ccmD*, (Schulz *et al*., 2000)] and heme lyase [*ccmF, ccmH* (Ren *et al*., 2000)] involved in heme uptake during synthesis of *c*-type cytochromes which are synthesized under anaerobic conditions (Iobbi-Nivol *et al*., 1994). Other genes involved in iron uptake such as *iroC* encoding an ABC transporter that exports the siderophore enterobactin (Crouch *et al*., 2008), *febA* (SENTW_ 2865) encoding an TonB-dependent outer membrane ferric enterobactin receptor and *fes* (SENTW_0562) encoding a Fes esterase that degrades siderophores to obtain free iron were transcribed in both samples but were significantly more transcribed in M9-glucose medium indicating an iron limitation or a high iron demand (Crouch *et al*., 2008).

Several genes involved in nitrogen uptake were upregulated in M9-glucose medium such as *glnK* (SENTW_0448) and *glnL* (SENTW_4090), which is a two-component system linked to glutamine utilization (Satomura *et al*., 2005) as well as *nirC* (SENTW_4222) encoding a probable nitrite transporter, and *nirB* and *nirD* (SENTW_4223–4224) encoding a nitrite reductase.

Besides higher expression of genes regulating uptake of nutrients such as iron, nitrogen and others, also genes for carbohydrate metabolism, biosynthesis of amino acids were more transcribed in M9-glucose medium (Table S1). Within this group, the majority of the higher transcribed genes represent the histidine biosynthesis operon *hisA-I* (SENTW_2197–2203). Several genes encoding hydrogenases were also more transcribed in M9-glucose medium such as *hypB-hypE* and *hybA-F* encoding hydrogenases. The Hyb proteins represent one of the three H_2_-consuming hydrogenases in *S.* Typhimurium (Zbell *et al*., 2007) containing NiFe centres (Lamichhane-Khadka *et al*., 2010). Genes of the *hyp* gene cluster encode a hydrogenase (*hypA-F*; SENTW_3276-81) that is, under fermentative growth conditions, regulated by a promoter localized within the *hypA* (SENTW_2942) gene. Both genes, as well as other single genes (Table S1) were significantly more transcribed in M9-glucose medium, indicating a potential anaerobic growth (Lutz *et al*., 2006).

### Influence of vegetable type on gene expression

Seven target genes (*cgsB*, *hutH*, *glpT*, *rcsA*, *sbp*, *nuoI* and *glnK*), identified by RNA-seq analyses as being significantly differentially transcribed and representing different functional categories, were selected for confirmation and further analysis by qRT-PCR. These selected genes had a high fold change in RNA-seq and might therefore play a significant role during the interaction of *S*. Weltevreden with plant material. In general, genes more transcribed in presence of sprouts analysed by RNA-seq were also more transcribed in presence of sprouts as determined by qRT-PCR, but the differences to M9-glucose medium were not always significant (Table 2). Comparison of expression ratios of qRT-PCR and fold change of RNA-seq analysis between the two sprouts samples (‘sprouts 48 h’ vs. ‘sprouts 48 h RNA-seq’) taken after 48 h showed similar results with fold changes in the same order of magnitude (Table 2).

**Table 2:** Transcription ratios of target genes, chosen for confirmation of RNA-seq analysis during growth with vegetables and in M9-glucose medium obtained. Transcription of mRNA was determined by quantitative reverse transcription-PCR. Fold change was determined using software REST which calculates whether genes are significantly (*P* < 0.05) upregulated (Up) or downregulated (Down). Fold changes without Up or Down behind numbers show no significant differences in expression between vegetables and M9-glucose medium

Target gene	Sprouts 24 h	Sprouts 48 h	Sprouts 48 h RNA-seq	Lamb's lettuce 24 h	Spinach 24 h	Lettuce 24 h
*csgB*	232.18 Up	873.47 Up	1520.48 Up	2750.99 Up	3154.95 Up	5289.82 Up
*glnK*	0.07 Down	0.54	0.38 Down	0.69	0.14 Down	1.62
*glpT*	3.30 Up	3.52	1.13	2.04 Up	16.24 Up	24.36 Up
*hutH*	1.64	6.02 Up	5.06 Up	9.15 Up	23.35 Up	11.40 Up
*nuoI*	0.45 Down	2.06	1.09	1.97 Up	4.66 Up	15.50 Up
*rcsA*	0.80	3.84 Up	1.26	2.28 Up	1.87 Up	33.09 Up
*sbp*	41.62	30.27	16.12 Up	29.17 Up	0.59	118.95 Up

Besides confirmation of RNA-seq results, influence of vegetable type was determined. Gene expression with sprouts was compared with *S*. Weltevreden grown on leafy salads such as lamb's lettuce, spinach and salad. Here, cells were harvested after 24 h because of decay of plant material afterwards, which caused the sample to contain too much plant material. Gene expression on leafy vegetables showed a significant upregulation of all target genes with one exception. The sulphate binding protein encoded by *sbp* (SENTW_4153) was not significantly more transcribed during growth with spinach. Comparing gene expression of *S*. Weltevreden in presence of sprouts after 24 and 48 h showed similar fold changes in the same order of magnitude with the exception of *hutH* and *rcsA*, both being significantly more transcribed after 48 h but not after 24 h.

One target gene (*glnK*) was chosen for analysis by qRT-PCR as RNA-seq analysis revealed it was solely transcribed in M9-glucose medium. Nevertheless, this gene was transcribed at low levels in presence of sprouts. Analysis by qRT-PCR showed no significant higher expression on vegetables but less significant expression with sprouts and spinach.

## Discussion

Interactions of human pathogens such as *Salmonella* spp. and *E. coli* O157:H7 with vegetables such as lettuce or alfalfa sprouts were analysed before. This study reports the complete transcriptome of a *Salmonella* spp. grown with alfalfa sprouts by RNA-seq analysis. So far, one microarray study analysed the transcriptome of *S.* Typhimurium SL1344 grown on cilantro leaves which was co-inoculated with *Dickeya dadantii*, a plant macerating pathogen (Goudeau *et al*., 2012) that showed a shift towards anaerobic metabolism. In two other microarray studies, transcriptome analyses of *E. coli* O157:H7 on vegetables have been reported (Kyle *et al*., 2010; Fink *et al*., 2012). The first studied the response of *E. coli* O157:H7 to lettuce lysate, resulting in strong oxidative stress of the bacterium. In the second recently published study, gene expression of *E. coli* O157:H7 on lettuce leafs was determined representing the first transcriptomic analysis of this pathogen on intact cell material (Fink *et al*., 2012). Both studies show similarities to our work, but gene expression patterns varied. This is most probably due to different plant material and to the use of *Salmonella* spp. as a pathogen in this study.

The initial step for establishment on plant tissue is attachment of bacteria to plant tissue (Brandl, 2006). In former studies, it was shown that curli and long aggregative fimbriae, which also were found to mediate binding to epithelial cells, were transcribed during attachment of *E. coli* O157:H7 to salad and of *Salmonella* spp. to alfalfa sprouts (Barak *et al*., 2005; Fink *et al*., 2012). In our study, the *csgDEFG* operon and *csgCAB* (*agfDEFG* and *agfCAB* equivalent) were transcribed in both media with *csgB, csgG* and *csgD* being more transcribed in presence of sprouts. The *csgDEFG* operon encodes for accessory proteins which are necessary for curli assembly while *csgD* encodes a positive transcriptional regulator for the *csgBA* operon [major curli subunit (Barnhart and Chapman, 2006)]. It was shown that *csgD* plays an important role in attachment of *S*. Newport to alfalfa shoots (Barak *et al*., 2005). Deletion of *csgB* reduced binding to alfalfa shoots during the first 24 h, whereas deletion of *csgA* had no effect (Barak *et al*., 2005). It was assumed that curli formation plays an important role at the first stage of plant colonization, which was also found for *E. coli* O157:H7 grown on lettuce (Fink *et al*., 2012). In our study, fold change for *csgB* was high, although samples were taken after 48 h, which represents a long inoculation period. A possible explanation might be that attachment to alfalfa sprouts was only starting at a later point during cultivation as the sample was slightly shaken during complete inoculation period. The sample contained both planktonic and attached cells. It might well be that cells, which lived planktonic during the first hours, started to attach to alfalfa sprouts later. Therefore, the *csg* operon was more transcribed in presence of sprouts only after 48 h. Besides the *csg* operon, the genes encoding BcfE, a fimbrin-like protein, and RcsA, a regulator for capsular polysaccharides, were more transcribed in presence of sprouts. Additional fimbriae and capsule production may indicate the importance of attachment of *S*. Weltevreden to sprouts after 48 h as both proteins enhance ability to attach to plant tissue (Hassan and Frank, 2004; Jeter and Matthysse, 2005).

Additionally, higher expression of the *fis*-encoded regulator was found in presence of sprouts. Fis regulates genes encoding the T3SS and its cognate effectors as well as synthesis of flagella for motility. Genes encoding flagella [*fli* and *flg* genes, (Barak *et al*., 2005; Jeter and Matthysse, 2005; Torres *et al*., 2005)] were not found to be more transcribed in presence of sprouts suggesting a more important role for Fis in regulation of the T3SS in our experiment. Indeed, genes of the T3SS encoded on SPI-2 were more transcribed in presence of sprouts, in contrast to growth in M9-glucose medium. Upregulation of several genes encoding proteins of the T3SS was found for *E. coli* O157:H7 grown in lysate of lettuce (Kyle *et al*., 2010) but not on lettuce leaves (Fink *et al*., 2012). SPI-2 plays the principal role during replication of intracellular bacteria within membrane-bound *Salmonella*-containing vacuoles (SCVs) in animal hosts (Cirillo *et al*., 1998). There are two possibilities for higher expression of genes encoding structural components of secretion machinery. First, they might be important for attachment to sprouts. Second, conditions in presence of sprouts might be similar to conditions as in SCVs inducing expression of SPI-2. As the sprout sample contained planktonic and attached cells, it remains unclear whether the cells induced these virulence genes as a stress response or for attachment on sprouts. Altogether, genes encoding only five of approximately 30 known effector proteins were found to be more transcribed in presence of sprouts. In the intestine of an infected host, the T3SS of SPI-1 is induced when cells come into contact with epithelial cells, seven effectors are translocated across host cell plasma membrane and membrane ruffling leads to invasion into the host (Galán, 2001; Patel and Galán, 2005). Several hours after uptake by host cells, an assembly of F-actin in close proximity to the SVC membrane and *Salmonella*-induced filaments (Sifs), which are induced by SPI-2 T3SS (Brumell *et al*., 2002), are released. At least 10 type III effectors are known to be associated with SCV encoded on SPI-2 (Heffron *et al*., 2011). As SPI-2 is only active after the bacteria reaches the intracellular vacuole, it might be more likely that the sprout environment mimics conditions found in the SCVs (Portillo *et al*., 1992; Rathman *et al*., 1996; 1997; Vescovi *et al*., 1996). The SCVs are characterized by an acidic pH and low nutrient concentrations such as Mg^2+^ (Cirillo *et al*., 1998; Beuzón *et al*., 1999; Löber *et al*., 2006): conditions that may also be found in the cultures with sprouts. Low expression of SPI-2 was also found for *S*. Typhimurium within a biofilm compared with planktonic cells because of environmental conditions (Hamilton *et al*., 2009). This might support the theory that part of SPI-2 was induced in presence of sprouts because of the environmental conditions. Alternatively, it might trigger the plant immune system (Schikora *et al*., 2011). In a recent study comparing plant and animal infection mechanisms, it was suggested that *Salmonella* spp. use translocation of effectors to remodel the host cells physiology to enhance entry to plant cell walls similar to animal tissue (Schikora *et al*., 2011). However, mechanisms of effectors delivery and the role of both SPI-1 and SPI-2 during plant infection remain unknown.

Besides attachment of single bacteria cells to plant cells, pathogens were found to attach at certain locations of the plant surfaces such as leaf veins and glandular trichomes (Monier and Lindow, 2005) and might build biofilms. Biofilm formation is a surface-associated growth (Hamilton *et al*., 2009), which might occur during growth of *S*. Weltevreden with sprouts. Hamilton and colleagues (2009) found that tryptophan and the *trp* operon are necessary for biofilm formation. This was also found for *E. coli* O157:H7 in the early stage of biofilm formation (Domka *et al*., 2007). However, in presence of sprouts, genes encoding the *trp* operon were transcribed, but expression was not significantly higher than in M9-glucose medium. It was found that *ssrA*, a regulatory gene encoded on SPI-2, plays a role in biofilm formation. Although this gene was transcribed under both conditions, it was not transcribed significantly higher in presence of sprouts. However, whether the SPI-2 T3SS plays an important role in biofilm formation remains unknown (Hamilton *et al*., 2009).

To establish on plant surface, pathogens have to adapt to an unfavourable habitat that is characterized by aerobic conditions, osmotic pressure, water stress and irregular distribution of nutrients on leave surfaces (Monier and Lindow, 2005). In contrast to leafy vegetables, sprouts might not represent those conditions. In our study, we rather found that *S*. Weltevreden cells showed a more transcribed set of genes required for sulphur metabolism as a possible reaction on low sulphur concentrations. These genes are mainly required for sulphate transport into cells and following reduction to sulphide. As plants are generally poor in sulphate, it was not surprising that *cys* regulon was more expressed in presence of sprouts. This was also found for *E. coli* O157:H7 grown on lettuce as well as on lettuce lysate (Kyle *et al*., 2010; Fink *et al*., 2012). Although growth conditions with sprouts differ from conditions found on leaves and lysate, demand for sulphur is given under all three conditions. For *E. coli* O157:H7, it was also found that phosphate starvation regulators *psiF* and *phoB* were more transcribed as well (Fink *et al*., 2012). This was not found in our study, and it allows the conclusion that the surface and exudates of alfalfa sprouts might not be poor in phosphate.

It was found that *S. enterica* preferentially colonize alfalfa roots (Anonymous, 2005), root hairs (Chapman *et al*., 1993) and in the mucilage close to the root tip (Veling *et al*., 2002). Root exudates consist mainly of mucilage (polysaccharides) and proteins (Evans *et al*., 1998). Several genes involved in carbohydrate metabolism were found to be more transcribed with only *cspB* and *rfbK* specific for mannose found on plants. We also found expression of three previously identified GIs specific for single *Salmonella* serovars encoding carbohydrate metabolism genes (Brankatschk *et al*., 2012). Here, it was shown that GI_IV was not transcribed in presence of sprouts and in M9-glucose medium whereas a low expression for GI_V was found and high expression for GI_VI (Brankatschk *et al*., 2012). It was assumed that GI_VI encoding a mannitol-specific PTS system might be specific for mannitol degradation. However, transcription of this GI is not significantly different between *S*. Weltevreden grown with sprouts and in M9-glucose medium. This GI might thus be specific to another carbon source other than mannitol. As an alternative carbon source to sugars, *S*. Weltevreden might use glycerol as well as fatty acids, as both systems were more transcribed in presence of sprouts. Another explanation might be that higher expression of genes for the glycerol uptake system and fatty acid metabolism is required for membrane generation.

Genes encoding stress response were found to be more transcribed in presence of sprouts. Highest expression has been found for single genes of the *psp* operon which usually is found during filamentous phage infection, mislocation of envelope proteins, extremes in temperature, osmolarity or ethanol concentrations and presence of proton ionophores (Darwin, 2005). Additionally, PspA might be an effector that plays a role in maintaining cytoplasmic membrane integrity (Darwin, 2005). As this operon is induced under several circumstances, it remains unclear why it is transcribed in presence of sprouts. It was also found to be transcribed on lettuce, and Fink and colleagues (2012) concluded that it was induced as a response to osmotic stress. Alternatively, it might play a role in biofilm formation as found for *E. coli* (Beloin *et al*., 2004) or that it is a response to surrounding environment as it was found to be transcribed during macrophage infection (Eriksson *et al*., 2003). A possible explanation might be osmotic stress because of the use of deionized water as inoculation matrix.

In our study, an additional gene *ipbA*, encoding a heat shock protein, and *osmY*, encoding a periplasmic protein, were more transcribed with sprouts, and both were found to be induced in *E. coli* during superoxide stress (Yim and Villarejo, 1992; Kitagawa *et al*., 2000). Injury of plant material is known to induce biochemical and signalling pathways in wound response such as production of an oxidative burst generating reactive oxygen. This might be a possible explanation for higher expression of *ipbA.* However, as sprouts were not cut or disrupted, it might not be a result of plant defence mechanism rather than using deionized water as an inoculation matrix that might also have led to induction of the *psp* operon.

For evaluation of RNA-seq analysis, seven genes were chosen for analysis of their transcription by qRT-PCR on alfalfa sprouts and additional vegetables. Results between a new sprouts sample taken after 48 h and the frozen RNA-seq sample were very similar with differences in the significance of expression ratios. Comparing gene expression analysed by qRT-PCR during growth on leafy vegetables to sprouts, it was shown that with leafy vegetables, expression ratios were higher than with sprouts. This might be explained by the fact that samples from leafy vegetables had to be taken already after 24 h because of leaf decay at 48 h. In a microarray study, it was found that fold change significantly varied over time and that it is dependent on the gene analysed (Kyle *et al*., 2010). Comparison of gene expression in presence of sprouts harvested after 24 h to leafy vegetables showed general lower expression ratios.

Comparing gene expression of sprouts sample taken after 24 and 48 h shows a shift in gene expression, which was also found by Kyle and colleagues (2010) and Fink and colleagues (2012). Because of adaption to the environment over time, there is a shift in the expression pattern of various metabolic pathways. Comparison of genes more transcribed with leafy vegetables showed similar results for significance of expression ratios except on spinach for the gene *sbp*, encoding the sulphate binding protein. A possible explanation might be that surface of spinach contains more sulphate than other vegetables, and therefore, genes encoding sulphate uptake might be less transcribed as found on other plants.

Growth of *S*. Weltevreden in M9-glucose medium showed genes more transcribed involved in nutrient uptake. In contrast to M9-glucose medium, genes involved in structuring siderophores, which have the capacity to chelate iron from the environment (Schaible and Kaufmann, 2004) and genes involved in heme storage were less transcribed in presence of sprouts. Heme-containing proteins are ubiquitous in nature (Daltrop *et al*., 2002). That and less expression of nitrogen regulatory proteins indicates that sprouts are rich in nutrients such as nitrate as well as iron in contrast to M9-glucose medium. Besides nutrient acquisition, higher transcription of genes encoding the synthesis of cytochromes and several genes encoding hydrogenases during growth in M9-glucose medium indicated anaerobic growth conditions. In a recent study, where the transcriptome of *S*. Typhimurium grown on cilantro was analysed (Goudeau *et al*., 2012), anaerobic growth conditions were also found on the plant. In their study, the cilantro was co-inoculated with *D. dadantii*, a pathogen macerating plant tissue, which could lead to more anaerobic conditions than on alfalfa sprouts as performed in this study.

With our study, we have shown that *S*. Weltevreden strain 2007-60-3289-1 adapts to the plant surface environment, which is characterized by extreme conditions but may be rich in root exudates including carbohydrates and proteins. For establishment, pathogens have to attach to plant tissue, which might be supported by generation of extracellular filaments known as curli. We confirmed expression of the *csg* operon encoding formation of curli known to be involved in the attachment on animal tissues. Here, *S.* Weltevreden strain 2007-60-3289-1 showed a similar colonization mechanism for the different plant tissues, as the *csg* operon was higher transcribed on both alfalfa sprouts and leafy vegetables. Higher transcription of five genes, encoding effector proteins and located on SPI-2, indicated that the sprout environment might be similar to conditions found in SCV during infection of animal tissue. Besides attachment mechanisms, *S.* Weltevreden strain 2007-60-3289-1 responded to sulphur stress with increased transcription of *cys* pathway for uptake of sulphur and following reduction. Less stress response-related genes compared with other studies were transcribed which might allow the conclusion that establishment on surface of sprouts is less characterized by stress factors regarding oxygen status, irregular distributed nutrients and osmotic stress, which is found in leafs. As we observed that *S*. Weltevreden strain 2007-60-3289-1 yielded larger cell pellets with sprouts than with fresh cut lettuce, it might be that sprouts represent a higher risk potential for infection by *Salmonella* spp. because of higher availability of nutrients than leafy and cut vegetables.

## Materials and methods

### Strains, growth medium and conditions

For total RNA extraction, *S*. Weltevreden strain 2007-60-3289-1 (Arthurson *et al*., 2010) was grown in liquid cultures of M9 minimal medium (Sambrook *et al*., 1989) with 10 mM glucose (M9-glucose medium) as sole carbon source and also with alfalfa (*Medicago sativa* L.) sprouts. In M9-glucose medium, cells were harvested during exponential growth (OD_600_ = 0.4) and diluted to OD_600nm_ of 0.1 (approximately 0.7 × 10^8^ cfu ml^−1^) for extraction. For sprouts cultures, strain *S.* Weltevreden 2007-60-3289-1 was pre-grown over night, washed and diluted to 10^6^ cfu ml^−1^ in sterile de-ionized water. Five-day-old alfalfa sprouts (1.5 g) were inoculated with 10 ml of this suspension. After 48 h at 21°C shaking at 40 r.p.m., culture liquid and sprouts were collected, vortexed and sonicated for 30 s. The sprouts were removed, culture liquid was centrifuged and the pellet was used for total RNA extractions. The sample contains therefore attached and planktonic cells that were collected during exponential phase. The pellet was shock-frozen in liquid nitrogen to ensure the status quo of cells at harvesting time until RNA extraction.

For verification of RNA-seq experiment, spinach (*Spinacia oleracea* L.), lamb's lettuce (*Valerianella locusta* L.) and leaf lettuce (*Latuca sativa* L., iceberg), alfalfa sprouts and M9-glucose medium were inoculated essentially identical as described above. For the experiment, 3 g of intact leaves were inoculated. Samples of spinach, lamb's lettuce and leaf lettuce as well as sprouts were taken after 24 h, since leaves were decayed after 48 h. Sprout samples were taken as well after 48 h as an independent sample for comparison with the samples used for RNA-seq analysis.

### Extraction of total RNA

Before total RNA extraction, pellets were treated with 100 μl of TE buffer containing 50 μg ml^−1^ lysozyme to enhance yield of total RNA. Extraction of total RNA from pellet of cultures grown in liquid medium was done using the NucleoSpin RNA II (Macherey-Nagel, Dueren, Germany). Extraction of total RNA from sprout supernatant was done using the innuPREP Plant RNA kit (Analytik Jena, Jena, Germany). After extraction, remaining DNA was removed using DNAse I (Fermentas, Thermo Scientific, Waltham, MA, USA) following the manufactures instructions. Presence of residual DNA was assayed by PCR using 16S rRNA gene-specific primers 63F and 1389R (Marchesi *et al*., 1998).

### cDNA libraries

Libraries for Illumina sequencing of cDNA were constructed by *vertis* Biotechnology AG, Freising, Germany (http://www.vertis-biotech.com/). For the sprouts sample, plant mRNA was separated first from bacterial RNA by removing the poly(A)-tail carrying RNA by oligo(dT) chromatography. Remaining RNAs were treated with Terminator exonuclease (TEX) to enrich bacterial primary transcripts carrying 5′-triphosphate. The transcripts resistant to TEX were fragmented by ultrasound treatment (four pulses of 30 s at 4°C), and with a poly(A) polymerase, poly(A) tails were added to the 3' ends of the RNA fragments. The polyadenylated RNA fragments were further treated with RNA-5′ polyphosphatase to remove 5′-triphosphate groups from the 5′ fragments. After ligation of a RNA oligonucleotide to the 5′ monophosphate of the RNA fragments, first-strand cDNA was synthesized using an oligo(dT)-linker primer and M-MLV H-reverse transcriptase. Finally, the cDNA was PCR-amplified using a high-fidelity DNA polymerase. Bacterial RNA was treated directly with TEX and the same procedure followed as for sprout sample. The purified cDNA samples were sequenced on an Illumina HiSeq 2000 machine to obtain 100 bp single end reads. For *S.* Weltevreden 2007-60-3289-1 grown in M9-glucose medium, 19 802 807 reads were generated by Illumina sequencing of the enriched cDNA library. For *S*. Weltevreden grown on alfalfa sprouts, 16 505 775 reads were sequenced.

### Mapping and statistical analysis

Reads were mapped against the draft genome sequence of *S.* Weltevreden 2007-60-3289-1 (Brankatschk *et al*., 2011) using Bowtie 2 (2.0.0-beta2) (Langmead *et al*., 2009). Generated SAM-files were transcribed into BAM-files using SAMtools (Li *et al*., 2009). For comparison of gene expression between *S*. Weltevreden grown with sprouts and in M9-glucose medium, BAM-files were compared using Cufflinks (1.2.0) (Trapnell *et al*., 2010). For each annotated gene, a value for FPKM (Fragments Per Kilobase of exon model per Million mapped fragments) was determined. For comparison of the two sample conditions, FPKM values for each gene were used to calculate a fold change. Significance of differently transcribed genes was after Benjamini-Hochberg correction of multiple testing. *P*-values lower than 0.05 were considered as significant.

For the sample M9-glucose, 8 758 337 reads (44.23%) could be aligned against the reference sequence of *S.* Weltevreden 2007-60-3289-1, whereas 11 044 470 reads (55.77%) failed to align. For the sample grown with sprouts, 6 352 340 reads (38.49%) aligned while 10 153 435 reads (61.51%) failed to align. In both cases, ineffective mRNA enrichment during depletion of ribosomal RNA before cDNA synthesis, the use of the incomplete genome sequence of S. Weltevreden 2007-60-3289-1 (Brankatschk *et al*., 2011) and the filtration of reads mapping on rRNA gene regions has influenced the mapping efficiency. Additionally, for the sprout sample, the lower number of mapping reads might be caused by the presence of RNA from the plant or from other bacteria that remained in the sample despite surface disinfection of seeds.

### Sequence analysis

The genome sequence of *S*. Weltevreden strain 2007-60-3289-1 consists of 66 contigs that were deposited in the EMBL database under accession numbers FR775188 through FR775253, and the plasmid pSW82 sequence was deposited under accession number FR775255 (Brankatschk *et al*., 2011). Additional BLAST searches were done at NCBI. Functions of genes of interest were classified according to the KEGG pathway database.

### RT-PCR and real-time quantification

Seven genes that were significantly differential transcribed (*P* < 0.05) between the two samples were selected for qRT-PCR to validate RNA-seq data and to test transcription on other vegetables. Primers were designed using *S*. Weltevreden 2007-60-3289-1 as a reference sequence (Table 3) with an amplicon size between 150 and 200 bp for each gene. Total RNA was extracted as described above.

**Table 3:** Primers designed for analysis of transcription ratios for target genes of *S*. Weltevreden 2007-60-3289-1 used for qRT-PCR

Primer^a^	Locus tag	Sequence (5′→3′)	Product size (bp)
csgB_F	SENTW_2110	TAATCAGGCGGCCATTATTGG	206
csgB_R		TATTACCGTAAGCGCTTTGCG	
hutH_F	SENTW_0770	TTGAGGGCACAGGAGTTATTTGC	194
hutH_R		ACAGTGGTGATGTGATTCAGC	
glpT_F	SENTW_2411	TTAACGACTGGAAAGCGGCG	178
glpT_R		TTCGCAGTCAGCTCTTCTTCC	
rcsA_F	SENTW_1101	AACCTGACTCGCTGGATACC	149
rcsA_R		AATCTGAATGGTTCCCTGACC	
sbp_F	SENTW_4153	TTACGATGTGGACGCTATTGC	175
sbp_R		GTAATCACCGACACACCGGG	
nuoI F	SENTW_2443	TTACCGTGGTCGTATCGTGC	219
nuoI R		AACTGAATCGCCGTGGTCGG	
glnK F	SENTW_0448	GGGAGGCGCTTTCTTCCATT	172
glnK_R		ATCACCTCTTCCAGTTGGTCG	
rpoD_F^*^	SENTW_3348	ACATGGGTATTCAGGTAATGGAAGA	61
rpoD_R^*^		CGGTGCTGGTGGTATTTTCA	
gmk F^*^	SENTW_3842	TTGGCAGGGAGGCGTTT	62
gmk R^*^		GCGCGAAGTGCCGTAGTAAT	

a Primers which were developed by (Botteldoorn *et al*., 2006) are indicated with an asterisk.

For RT-PCR, the RevertAid H Minus First Strand cDNA Synthesis Kit (Fermentas, Thermo Scientific, Waltham, MA, USA) and random hexamer reverse primers were used following the manufactures instructions. Amplification of gene transcript was performed on the ABI Prism 7500 Sequence detection system (Applied Biosystems Europe BV, Zug, Switzerland). All reactions were performed with the Kapa SYBR Fast qPCR Universal Kit (Kapa Biosystems, Cape Town, South Africa). For data normalization, two housekeeping genes *rpoD* and *gmk* (Botteldoorn *et al*., 2006) were used as an internal reference to obtain more reliable basis of normalization (Pfaffl *et al*., 2002). All experiments were done in three independent replicates and additionally three replications within each qRT-PCR run. Fold change between vegetable sample and M9-glucose medium was calculated using relative expression software REST (Pfaffl *et al*., 2002).

## Supporting information

**Table S1.** Genes higher transcribed in M9-glucose medium in comparison to sprouts determined by RNA-seq analysis.
