# Supplementary material for: Transcriptional profile of Salmonella enterica subsp. enterica serovar Weltevreden during alfalfa sprout colonization
Source: Microb Biotechnol. 2013 Dec 6;7(6):528–44. doi: 10.1111/1751-7915.12104 (PMC4265072; doi:10.1111/1751-7915.12104)
Supplement: Supplementary file 1 — Table S1. Genes higher transcribed in M9-glucose medium in comparison to sprouts determined by RNA-seq analysis. [file mbt20007-0528-sd1.pdf]

Supplemental Data to:

K. Brankatschk, Tim Kamber, Joël F. Pothier, Brion Duffy and Theo H.M. Smits

Transcriptional Profile of *Salmonella enterica* subsp. *enterica* Serovar Weltevreden during Alfalfa Sprout Colonization

Table S1. Genes higher transcribed in M9-glucose medium in comparison to sprouts determined by RNA-seq analysis.

Category	Gene	Locus tag	Fold change	Function
Amino acid metabolism	*lysC*	SENTW_4304	26.36	Aspartokinase III
	asnA	SENTW_3982	23.72	Asparagine synthetase A
	skd	SENTW_3964	12.09	Shikimate 5-dehydrogenase
	araD	SENTW_0054	9.87	L-ribulose-5-phosphate 4-epimerase
	ilvC	SENTW_4005	9.86	Ketol-acid reductoisomerase
	tyrA	SENTW_2788	8.94	Chorismate mutase-T and prephenate dehydrogenase
	aroF	SENTW_2789	8.75	Phospho-2-dehydro-3-deoxyheptonate aldolase
	thrB	SENTW_4706	6.22	Homoserine kinase
	ordL	SENTW_3257	5.14	Putative oxidoreductase
	pmrF, pqaB, yfbG, yfbH, yfbJ, yfbW	SENTW_2421-2426	3.37	Putative sugar transferase, formyltransferase, polysaccharide deacetylase, transferase, hypothetical protein, hypothetical protein
	trpA, trpB	SENTW_1471-1472	3.31	Tryptophan synthase
	ilvI, ilvH	SENTW_0072-0073	3.18	Acetolactate synthase
	hisA, hisC, hisB, hisH, hisF, hisI	SENTW_2197-2203	3.1	Isomerise, aminotransferase, dehydratase, phosphate synthase subunit, isomerise, phosphate synthase subunit, pyrophosphatase protein
Pathogenicity	*srfC, srfB1. srfB2*	SENTW_1611-1613	4.01	Putative virulence factor
	invE, invG, invF	SENTW_2986-2988	4.35	Invasion protein
	hofC,outE	SENTW_0101-0102	5.69	Type IV pilin biogenesis protein, conserved protein with nucleoside triphosphate hydrolase domain
	sipC	SENTW_2973	8.78	Effector protein
	yopP	SENTW_2953	9.75	Secreted effector protein
Motility	*fimA*	SENTW_0142	37.89	Putative fimbrin-like protein
	fliK, fliJ	SENTW_1108-1109	12.04	Flagellar protein; Otogelin Flags
	fimH	SENTW_4729	6.33	Uncharacterized fimbrial-like protein
	stbC, stbB	SENTW_0321-0322	4.65	Fimbrial chaperon protein, Outer membrane usher protein
	cheR , cheB	SENTW_1164-1163	6.75	Protein-glutamate methylesterase; chemotaxis protein methyltransferase
	cheA	SENTW_1160	5.46	Chemotaxis protein
	ygiX,ygiY	SENTW_3311-3312	5.18	Transcriptional regulatory protein, sensor histidine kinase

	tar	SENTW_1162	4.07	Methyl-accepting chemotaxis protein II, aspartate sensor receptor
	yfcS	SENTW_0164	15.72	Pili chaperone protein
Cofactors and energy production	*yfiD*	SENTW_1232	4.32	Autonomous glycyl radical cofactor
	ygbK, pdxA3, kdgT	SENTW_0126-0128	6.61	2-keto-3-deoxygluconate permease, putative inner membrane protein, 4-hydroxythreonine-4-phosphate dehydrogenase
	ynhD, ynhC, sufS	SENTW_1840-1842	3.85	Cysteine desulfurase/selenocysteine lyase, desulfurase activator, desulfurase ATPase
	panE	SENTW_2762	6.36	2-dehydropantoate 2-reductase
	cbiB, *cbiA*	SENTW_2158-2159	5.51	Cobyrinic acid A,C-diamide synthase
Regulators	SENTW 3738, *celR*	SENTW_3738, 2840	30.52	Regulatory protein
	prpR	SENTW_0349	13.6	Propionate catabolism operon regulatory protein
	sprB1	SENTW_2954	7.7	Transcriptional regulator
	xapR1	SENTW_2598	6.99	Transcriptional regulator
	treR	SENTW_4560	6.16	Transcriptional Regulator
	hilD1	SENTW_2964	5.48	Transcriptional regulator
	yhjB	SENTW_3706	5.34	Transcriptional regulator
	fhlA	SENTW_2947	4.84	Formate hydrogenlyase transcriptional activator
	budR	SENTW_2764	4.66	Transcriptional regulator
	ywbI	SENTW_4356	4.53	Probable RuBisCO transcriptional regulator
	alcR	SENTW_0345	4.45	HTH-type transcriptional activator
	hilD3	SENTW_2955	4.34	Transcriptional regulator
	glnL	SENTW_4090	3.95	Nitrogen regulation protein
	basR	SENTW_4379	3.82	DNA-binding transcriptional regulator
		SENTW_4300	2.52	DeoR family regulatory protein
	srlR,	SENTW_2924	2.38	DNA-bindng transcriptional repressor
	gutM	SENTW_2923	6.05	Glucitol operon activator protein
	caiF	SENTW_0019	5.74	Transcriptional activatory protein
	citB, citA	SENTW_4760-4761	5.21	Response regulator, sensor kinase (Citrate fermentation)
	yegO3, mdtC, yegB, baeS, baeR	SENTW_2253-2257	4.24	Multidrug efflux system subunit, signal transduction histidine-protein kinase, DNA-binding transcriptional regulator
Transporters	*ail*	SENTW_0330	∞	Outer membrane protein
	gatA	SENTW_3389	∞	PTS system
	yjfI	SENTW_4477	∞	inner membrane protein
	iroC	SENTW_2862	20.15	Multidrug resistance protein

	psaA, CYSA,sitC,sitD	SENTW_2949-2952	13.47	ABC transporter, Chelated iron transport
	ompC	SENTW_2395	12.11	Putative outer membrane
	pucJ	SENTW_3736	9.35	Putative xanthine permease
	cyaB,cyaD,srpC	SENTW_2856-2858	9.32	Putative type I secretion protein
	nirC	SENTW_4222	8.87	Probable nitrite transporter
	yfgF	SENTW_2686	8.75	Innermembrane protein
	sfbA	SENTW_0496	7.82	Lipoprotein
	nmpC	SENTW_1635	7.65	Outer membrane pore protein
	yjfL	SENTW_4477	7.26	Inner membrane protein
	ydiM	SENTW_1851	7.19	Inner membrane transport protein
	nhaC	SENTW_1654	6.99	Uncharacterized Na(+)/H(+) antiporter
	artJ	SENTW_4458	6.45	Arginine-binding periplasmic protein
	ptlG	SENTW_4420	5.64	Putative multidrug resistance protein
	sfbB,sfbC	SENTW_0497-0498	5.33	Methionine import ATP-binding protein, lipoprotein
	yebL	SENTW_1193	5.26	High-affinity zinc uptake system protein znuA
	ydjN1	SENTW_1845	5.12	Putative L-cystine uptake protein
	ybbL,ybbM	SENTW_0488-0489	5.09	Spermidine/putrescine import ATP-binding protein, innermembrane protein
	yojI	SENTW_2391	4.9	Lipid A export ATP-binding/permease protein
	modA,modB	SENTW_0755-0756	4.81	Molybdate-binding periplasmic protein
	yceE	SENTW_0365	4.53	Multidrug resistance protein
	yahN	SENTW_0347	4.31	Putative threonine efflux protein
	yjgN	SENTW_4583	4.21	Inner membrane protein
	exbB	SENTW_3291	4.18	Biopolymer transport protein
	tet	SENTW_4826	4.13	High-affinity glucose transporter
	kefB	SENTW_4243	4.12	Efflux system protein
	tehA, theB	SENTW_1596-1597	3.89	Tellurite resistance/ dicarboxylate transporter
	znuB, znuC	SENTW_1191-1192	3.7	Zinc import ATP-binding protein
	sgaT	SENTW_4486	5.08	PTS system
	yejB, dppC	SENTW_2351-2352	3.2	Permease
	yadI	SENTW_0143	4.3	Putative PTS system IIA component
	fruA3	SENTW_2336	7.93	PTS system, fructose-specific IIBC component
	kdgT	SENTW_0632	5.26	2-keto-3-deoxygluconate permease 2
Lipid metabolism	*dacD*	SENTW_2186	10.64	DD-carboxypeptidase
	pduP	SENTW_2175	16.97	Retinal dehydrogenase

	ppiC	SENTW_4006	11.08	Peptidyl-prolyl cis-trans isomerase C
Carbohydrate metabolism	*fucA*	SENTW_3083	∞	L-fuculose-1-phosphate aldolase
	fucK	SENTW_3086	5.32	L-fuculokinase
	rhaD	SENTW_4132	5.28	Rhamnulose-1-phosphate aldolase
	citF, citX ,citG	SENTW_0010-0012	4.78	Citrate lyase alpha chain, holo-ACP synthase, triphosphoribosyl-dephospho-CoA synthase
	yrbE	SENTW_4537	33.45	Oxidoreductase
	pcm, surE, truD, ygbO, ygbP	SENTW_3013-3017	2.26	Protein-L-isoaspartate(D-aspartate) O-methyltransferase, stationary phase survival protein, tRNA pseudouridine13 synthase, 2-C-methyl-D-erythritol 2,4-cyclodiphosphate synthase, 2-C-methyl-D-erythritol 4-phosphate cytidylyltransferase
	galM, galK	SENTW_0747-0748	2.92	Galactokinase
	gutQ	SENTW_2925	3.48	D-arabinose 5-phosphate isomerase
	gatZ	SENTW_3388	3.99	Putative tagatose 6-phosphate kinase 1
	adhE	SENTW_1446	7.92	Aldehyde-alcohol dehydrogenase
	fruK , fruB	SENTW_2337-2338	7.4	PTS system, fructose-specific IIA component, fructose-1-phosphate kinase
	yhcJ	SENTW_1049	∞	Putative N-acetylmannosamine-6-phosphate epimerase
	yafB	SENTW_0216	7.26	2,5-diketo-D-gluconate reductase B
Nucleotide metabolism	*purE, purK*	SENTW_0518-0519	3.77	Carboxylase, hydrolase
	allD	SENTW_0513	13.87	Ureidoglycolate dehydrogenase
Genetic information processing				
– Replication and repair	*ftsY1*	SENTW_3662	5.33	Cell division protein
	ykgM	SENTW_0454	30.63	50S ribosomal protein
Phage genes	U 50	SENTW_0968	∞	Probable tail fiber assembly protein
	SENTW_2536	SENTW_2536	∞	Bacteriophage
	SENTW_2553	SENTW_2552-2553	∞	Salmonella phage
	V, W	SENTW_2822-2823	∞	Phage
	SENTW 2831,gpT1,gpT3	SENTW_2831-2833	965.42	Family phage tail tape measure protein
	FI	SENTW_2828	7.05	Major tail sheath protein
	B,sid	SENTW_4602-4603	6.66	Capsid morphogenesis protein from phage; Late control gene B protein
	SENTW 2808, P	SENTW_2808-2809	5.47	2
	K, I	SENTW_1285-1286	4.71	Tail assembly protein K, tail assembly protein
	J	SENTW_1287	4.39	Host specificity protein

	nohA, 2, *vhtJ*, 4a, 4b, 5 6	SENTW_1203-1208	3.01	Gifsy-1 prophage DNA packaging protein, terminase, Gifsy-1 prophage head to tail joining protein, head-tail preconnector-like protein, head-tail preconnector protein GP5 [contains: scaffold protein GP6 (head protein GP6)]
	5, 8	SENTW_2531-2532	2.96	Coat protein, scaffolding protein
Anaerobic metabolism	*ccmC, ccmD, ccmE, ccmF, ccmG, ccmH*	SENTW_2375-2380	∞	Heme exporter
	hybF, hybE, hybD, hybC, hybB, hybA	SENTW_3276-3281	3.99	Hydrogenase nickel insertion, hydrogenase 2-specific chaperone, endopeptidase , hydrogenase subunits
	hypB,hypC,hypD,hypE	SENTW_2943-2946	5.6	Hydrogenase nickel incorporation protein, Hydrogenase isoenzymes formation protein, Hydrogenase isoenzymes formation protein, hydrogenase expression/formation protein
	fdhF1	SENTW_4371	5.33	Formate dehydrogenase H
	dmsC	SENTW_1716	4.77	Anaerobic dimethyl sulfoxide reductase chain
	dmsA	SENTW_4391	4.3	Anaerobic dimethyl sulfoxide reductase subunit A
	hydH	SENTW_4281	8.45	Sensor kinase
	hypA	SENTW_2942	5.99	Hydrogenase nickel incorporation protein
Iron metabolism	*fes*	SENTW_0562	66.91	Enterochelin esterase
	fepA	SENTW_2865	22.68	Ferric enterobactin receptor
	feoA	SENTW_3601	6.99	Ferrous iron transport protein A
	fixC,fixX	SENTW_0029	27.7	Putative oxidoreductase; Ferredoxin-like protein
	iroB	SENTW_2861	13.07	Putative glucosyltransferase
Nitrogen/sulfur metabolism	*glnK*	SENTW_0448	∞	Nitrogen regulatory protein P-II
	nirD, nirB	SENTW_4223-4224	4.13	Nitrite reductase (NAD(P)H)
	cysG	SENTW_4221	8.06	Siroheme synthase
	ttrC ,ttrA	SENTW_1829-1830	6.55	Tetrathionate reductase
	torD, yyaE, dmsB, ynfH	SENTW_0587-0590	2.56	Putative component of anaerobic dehydrogenase, Periplasmic nitrate reductase (molybdopterin oxidoreductase), molybdopterin-containing oxidoreductase iron sulfur subunit, molybdopterin-containing oxidoreductase membrane anchor subunit
	dapB	SENTW_0013	6.45	Dihydrodipicolinate reductase
Exoenzyme	SENTW 0381,*sbcC,sbcD*	SENTW_0379-0381	48.08	Exouclease subunit
	ycfO	SENTW_2044	6.58	Beta-N-acetylhexosaminidase
	exoO	SENTW_2568	5.11	Endodeoxyribonuclease
Unclassified	*crcB*	SENTW_0609	8.89	Camphor resistance protein
	SENTW_2009	SENTW_2009	∞	putative oxidoreductase

	SENTW 2697,*ratA1*	SENTW_2697-2698	3472.88	
	ybhA, SENTW_4094, *hemN3*	SENTW_4093-4095	3.44	Hydrolase, hypothetical protein, coproporphyrinogen III oxidase
	yjbF, yjbG, yjbH	SENTW_4308-4310	3.82	Uncharacterized lipoprotein, hypothetical protein, putative lipoprotein
	degS	SENTW_3478	8.72	Serine protease
	yqcB,yqcC	SENTW_3074-3075	7.13	Putative RNA pseudouridylate synthase, Hypothetical protein
	yghU	SENTW_3272	6.12	Putative glutathione S-transferase
	hsdR	SENTW_4636	6.01	Type I restriction-modification system
	ydfZ	SENTW_1702	5.64	Putative selenoprotein
	yjdB	SENTW_1576	2.53	Putative metal dependent hydrolase
	yrfG	SENTW_3592	4.94	Putative hydrolase of the HAD superfamily
	yaiV	SENTW_0357	4.91	Putative DNA-binding transcriptional regulator
	ydiF	SENTW_1856	4.53	Coenzyme A transferase
	yaiH	SENTW_0358	4.5	Penicillin-binding protein
	traI, traD	SENTW_4777-4778	4.47	Conjugal transfer protein, conjugal transfer nickase/helicase
	yggA	SENTW_3182	4.37	Arginine exporter protein
	phoN	SENTW_4403	4.22	Nonspecific acid phosphatase precursor
	SENTW 5538,*insD*	SENTW_5538-5539	4.2	Transposase
Hypothetical proteins	*ycgK*	SENTW_1202	∞	Uncharacterized protein
	ydjL	SENTW_1927	∞	hypothetical protein, only in Choleraesius str. SC-B67
		SENTW_1197-1198	80.26	Hypothetical protein, Hypotethical phage protein
		SENTW_1978	44.79	Hypothetical protein
	yodA	SENTW_1965	20.52	Hypothetical protein
		SENTW_1608	20.15	Hypothetical protein
	ygaW	SENTW_2890	20.15	Hypothetical protein
	SENTW 1610,*ydcX*	SENTW_1609	20.03	Hypothetical protein
	Mmab	SENTW_2174	16.97	Hypothetical protein
		SENTW_4395	15.97	Hypothetical protein
	yrfB,yrfC,yrfD	SENTW_3584-3586	15.84	Hypothetical protein
	yhhT	SENTW_3675	14.11	Hypothetical protein
		SENTW_0014	13.3	Hypothetical protein
		SENTW_5520	12.61	Hypothetical protein
	ybeD	SENTW_0616	12.19	Hypothetical protein
		SENTW_1247	11.29	Hypothetical protein

	SENTW_4598-4600	10.99	Hypothetical protein
	SENTW_4419	10.28	Hypothetical protein
	SENTW_4620	10.08	Hypothetical protein
	SENTW_3740	9.87	Hypothetical protein
yaeQ, yaeJ	SENTW_0206-0207	9.53	Hypothetical protein, peptidyl-tRNA hydrolase domain protein
yddG	SENTW_1637	9.51	Hypothetical protein
creD	SENTW_4695	9.33	Hypothetical protein
yhjU, yhjT, yhjS	SENTW_3725-3726	9.23	Hypothetical protein
	SENTW_3729		
yjiJ	SENTW_4626	8.64	Hypothetical protein
	SENTW_1531	8.6	Hypothetical protein
yfeN	SENTW_2602	8.5	Hypothetical protein
	SENTW_3286	8.06	Hypothetical protein
yfcC	SENTW_4571	8.06	Hypothetical protein
	SENTW_2995	7.72	Hypothetical protein
yaaI	SENTW_4714	7.52	Hypothetical protein
	SENTW_1331	7.26	Hypothetical protein
	SENTW_3025	7.32	Hypothetical protein
	SENTW_4303	7.05	Hypothetical protein
ybhM	SENTW_0788	6.99	Hypothetical protein
SENTW 2784,*yfiO*	SENTW_2783-2784	6.68	Hypothetical protein; putative outer membrane protein
	SENTW_1058	6.31	Hypothetical protein
	SENTW_2125	6.13	Hypothetical protein
yjcC	SENTW_4348	6.09	Hypothetical protein
	SENTW_1634	6.05	Hypothetical protein
	SENTW_4302	6.05	Hypothetical protein
ydiZ	SENTW_1887	5.91	Hypothetical protein
	SENTW_4617	5.84	Hypothetical protein
	SENTW_0093	5.78	Hypothetical protein
	SENTW_1142	5.69	Hypothetical protein
ypeC	SENTW_2590	5.64	Hypothetical protein
yjbQ	SENTW_4335	5.49	Hypothetical protein
	SENTW_0631	5.44	Hypothetical protein
ynjA	SENTW_2894	5.44	Hypothetical protein

		SENTW_5514	5.33	Hypothetical protein
	iroE	SENTW_2864	5.28	Hypothetical protein
		SENTW_1529	5.14	Hypothetical protein
	yqfB	SENTW_3164	4.97	Hypothetical protein
		SENTW_4346	4.89	Hypothetical protein
		SENTW_4739	4.88	Hypothetical protein
	dedD,folC	SENTW_2490-2491	4.85	Hypothetical protein, dihydrofolate:folylpolyglutamate synthetase
		SENTW_1884	4.84	Hypothetical protein
	ygaC	SENTW_2891	4.71	Hypothetical protein
		SENTW_3623	4.69	Hypothetical protein
		SENTW_3868	4.64	Hypothetical protein
	yczH	SENTW_0306	4.57	Hypothetical protein
	yeaJ	SENTW_1942	4.55	Hypothetical protein
	trpH	SENTW_1476	4.55	Hypothetical protein
	iicA	SENTW_4625	4.47	Hypothetical protein
	ppdC, ygdB, ppdB, ppdA	SENTW_3106-3109	4.44	Hypothetical proteins
		SENTW_0483	4.43	Hypothetical protein
		SENTW_1250	4.43	Hypothetical protein
	ynfB	SENTW_1709	4.35	Hypothetical protein
		SENTW_0033	4.34	Hypothetical protein
		SENTW_2500	4.32	Hypothetical protein
		SENTW_3902	4.27	Hypothetical protein
		SENTW_2720	4.22	Hypothetical protein
	yfeD	SENTW_2595	4.19	Hypothetical protein
		SENTW_4301	3.79	Hypothetical protein
	yqaA, yqaB	SENTW_2911-2912	2.78	Hypothetical protein, fructose-1-phosphatase
	CP83,SENTW 2802,*exoX*	SENTW_2800-2802	5.4	Putative exonuclease, Hypothetical protein, Hypothetical protein
